# Effect of Gd Content on the Microstructure and Mechanical Properties of Hot Extruded Mg-*x*Gd-4Y-1Sm-0.5Zr Alloys

**DOI:** 10.3390/ma18215023

**Published:** 2025-11-04

**Authors:** Lipeng Yan, Xinglin Zhu, Ranfeng Qiu, Nannan Wang, Xiaoke Zhang

**Affiliations:** 1 College of Material Science and Engineering, Henan Institute of Technology, Xinxiang 453003, China; zhuxl@hait.edu.cn (X.Z.);; 2 Henan Key Laboratory of Advanced Cable Materials and Intelligent Manufacturing, Henan Institute of Technology, Xinxiang 453003, China; 3 School of Materials Science and Engineering, Henan University of Science and Technology, Luoyang 471039, China; qiurf1221@163.com; 4 Luoyang College, Civil Aviation Flight University of China, Luoyang 471000, China

**Keywords:** Mg-*x*Gd-4Y-1Sm-0.5Zr, hot-extruded alloy, bimodal microstructure, aging, fracture

## Abstract

In this paper, the microstructure, mechanical properties, and strengthening mechanisms of hot-extruded Mg-*x*Gd-4Y-1Sm-0.5Zr (*x* = 4, 7, 10, wt.%) alloys were studied. The results show that the hot extruded alloys exhibit bimodal grain structures, and with Gd content increasing, the fraction of non-dynamic recrystallized grains gradually decreases, with 46.3%, 38.6%, and 9.3%. After aging for 200 °C × 96 h, all three hot-extruded alloys reach peak-aged hardness, and as Gd content increases, the area number density of the β′ phase increases with Gd increasing, being 7.1 × 10^15^/m^2^, 9.9 × 10^15^/m^2^, and 16.5 × 10^15^/m^2^, respectively. And the yield strength (YS) increases from 287 MPa to 345 MPa, the ultimate tensile strength (UTS) increases from 365 MPa to 418 MPa, and elongation (EL) decreases from 8.5% to 4.2%. The tensile failure mechanism is quasi-cleavage fracture. With Gd content increasing, the dimples and tear ridges on fracture surfaces gradually decrease while cleavage facets increase. The peak-aged GWS741 alloy demonstrates optimal comprehensive mechanical properties, with YS, UTS, and EL reaching 332 MPa, 409 MPa, and 7.8%, respectively. During in situ tensile testing, coarse un-DRXed grains undergo prismatic ($\{10\overset{-}{1}0\}\langle 11\overset{-}{2}0\rangle$) slip, while DRXed grains experience basal ($\left\{0001\right\}\langle 11\overset{-}{2}0\rangle$) slip and twinning deformation. Even at 6.6% strain, no microcracks are observed, indicating excellent plasticity. During the tensile failure process, the main crack propagates along tortuous paths, showing crack deflection characteristics, where it either penetrates through elongated deformed grains or bypasses un-DRXed grains.

## 1. Introduction

As the lightest metallic structural material, Mg alloys have demonstrated significant application value in fields such as new energy vehicles, military equipment, and the aerospace field. Among them, Mg-Gd-Y series alloys are a hot spot due to the high room-temperature and high-temperature mechanical properties [1,2,3]. This is because the atomic radii of gadolinium (Gd) and yttrium (Y) differ significantly from that of Mg, resulting in a remarkable solution strengthening [4], and another reason is that the maximum solid solubilities of Gd and Y in the Mg matrix are 23.5 wt.% and 12.8%, respectively, and their solubilities vary significantly with the temperature, providing a basis for age hardening [5,6]. Studies show that hot deformation can refine grains and improve the mechanical properties of Mg-Gd-Y alloys [7,8,9]. Alizadeh et al. [10] found that the hot deformation Mg-5Gd-4Y-0.4Zr alloy exhibited remarkable thermal stability, thereby significantly enhancing the alloy’s high-temperature strength. Wan et al. [11] prepared nanocrystalline Mg-8Gd-3Y-0.4Zr alloys by rotary swaging method. Dobatkin et al. [12] also obtained a nanocrystalline Mg-4.6Gd-4.7Y-0.3Zr alloy via high-pressure torsion. Compared with the undeformed state, the strength of the alloy was significantly enhanced. Zheng et al. [13] achieved uniformly distributed dynamically precipitated phases through pre-aging treatment and thus prepared a high-strength hot-extruded Mg-9.5Gd-4Y-2.2Zn-0.5Zr alloy.

Sm element can refine grain and promote the aging precipitation of rare earth atoms, further improving mechanical properties in Mg-Gd-Y alloys [14,15]. Studies on Mg-Gd-Y-Sm-Zr alloys have begun to receive significant attention from researchers at the moment. Yan et al. [16] successfully prepared a high-temperature-resistant Mg-8Gd-4Y-1Sm-0.5Zr alloy and found that the precipitates effectively suppressed grain boundary sliding during high-temperature tensile deformation. Pei et al. [17] researched the microstructure evolution of Mg-Gd-Y-Sm-Zr alloy and focused on analyzing the dynamic recrystallization mechanism using a hot compression test. Li et al. [18] investigated the influence of Sm addition on the microhardness of the Mg-12Gd-2Y-0.5Zr alloy, observing an increase from 121.4 HV to 134.4 HV, which confirmed the solid-solution strengthening effect of Sm.

The above research mainly focuses on the dynamic recrystallization mechanism and deformation mechanism of Mg-Gd-Y-Sm-Zr alloy. However, there has been limited investigation into the impact of Gd content on the alloy’s mechanical properties, and research on optimizing the Gd element content is also lacking. In this paper, gradient Mg-*x*Gd-4Y-1Sm-0.5Zr (*x* = 4, 7, 10, wt.%) alloys were designed. Combined with characterization techniques including scanning electron microscopy, electron backscatter diffraction, and high-angle annular dark-field scanning transmission electron microscopy, the microstructure, mechanical properties, and fracture morphology were characterized. The research focused on the microstructural evolution, strengthening-toughening mechanisms, and deformation mechanisms, aiming to provide a theoretical basis for exploring microstructural evolution laws and controlling the mechanical properties of hot-extruded Mg-Gd-Y-Sm-Zr alloys.

## 2. Materials and Methods

The Mg-*x*Gd-4Y-1Sm-0.5Zr (*x* = 4, 7, 10; wt.%) alloys were fabricated by gravity casting technology in our laboratory. The melting process involved placing pure magnesium (99.9%), Mg-Gd (30%), Mg-Y (30%), Mg-Sm (30%), and Mg-Zr (30%) (wt.%) alloys into a crucible. Simultaneously, a mixture of CO_2_ and SF_6_ gases (99:1) was introduced as a protective atmosphere. The raw materials were heated to 760 °C, held for 10 min, and then poured into a steel mold, forming an as-cast ingot. The specific components are shown in Table 1. As-cast alloys were subsequently subjected to solution treatment at 525 °C × 10 h. The solution-treated alloys were subjected to hot extrusion forming with an extrusion temperature of 500 °C, an extrusion ratio of 12, and an extrusion speed of 5 mm/s. Finally, the hot-extruded specimens were aged at 200 °C, and the hardness and tensile properties were tested.

The microstructures of the as-cast, solution-treated, and extruded experimental alloys were characterized using an optical microscope (OM, YR300, Hefei, China) and a field emission scanning electron microscope (SEM, JSM-5610LV, JEOL, Tokyo, Japan) equipped with an Oxford HKL Channel 5 electron backscatter diffraction (EBSD, JSM-7800F, JEOL, Tokyo, Japan) detector. The precipitated phases were characterized by an transmission electron microscope (TEM, FEI Titan 80-300, Waltham, American). The aging hardness of the alloys was measured by the Vickers hardness tester (HVS-1000Z, Suzhou, China). The mechanical properties were tested by a microelectronic universal testing machine (AG-1250KN, Shimadzu, Tokyo, Japan) and three tests were conducted on each alloy sample.

## 3. Results and Discussions

### 3.1. As-Cast and Solid Solution Microstructures

**Figure 1:**
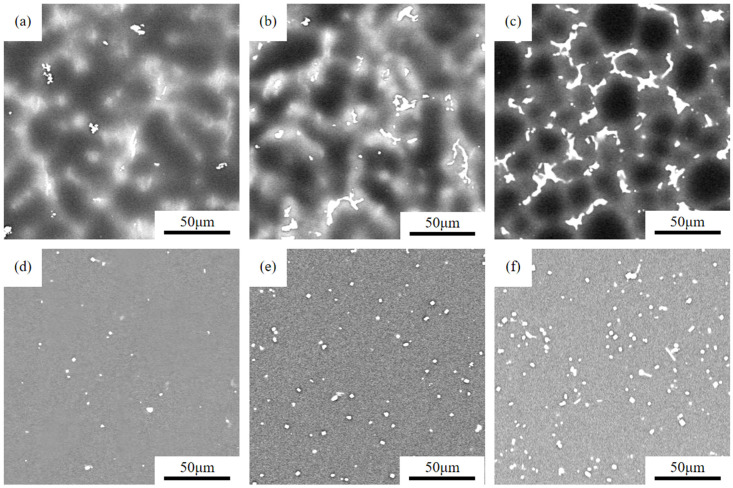
As-cast (**a**–**c**) and solid solution SEM images (**d**–**f**) of Mg-*x*Gd-4Y-1Sm-0.5Zr alloys. (**a**,**d**) *x* = 4; (**b**,**e**) *x* = 7; (**c**,**f**) *x* = 10.

Figure 1a–c show SEM images of as-cast Mg-*x*Gd-4Y-1Sm-0.5Zr (*x* = 4, 7, 10; wt.%) alloys. It is evident that the microstructure of as-cast GWS441 alloy consists of α-Mg matrix and white second phases distributed in the figures. The second phase, which is small in quantity and size, is mainly blocky and elongated, presenting a dispersed distribution. With Gd increasing, the quantity of second phases increases significantly. When the Gd content is 10% (Figure 1c), the second phases exhibit an intermittent network distribution. This is because during the casting process of the alloy, the cooling rate of the melt is too fast, leading to insufficient diffusion of rare earth elements such as Gd, Y, and Sm, which tend to accumulate at the grain boundaries and precipitate in the form of second phases [19].

Figure 1d–f present the SEM images of the solution-treated alloy microstructure. After solution treatment, most of the second phases in the as-cast microstructure have been dissolved into the matrix, with only a small amount of block (dot-like) undissolved phases remaining. The research shows that these undissolved particles are Mg_5_(Gd,Y,Sm) phases [20]. With the increase in Gd content, the amount of undissolved phases increases gradually.

**Figure 2:**
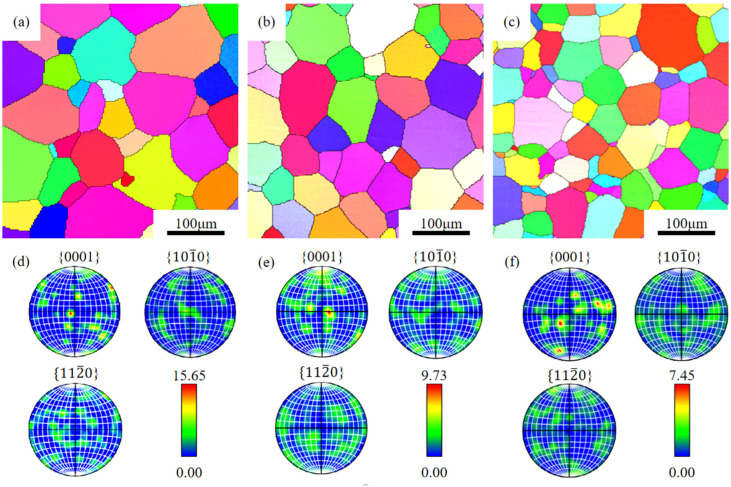
IPF maps (**a**–**c**) and the corresponding PF maps (**d**–**f**) of the solid-solution Mg-*x*Gd-4Y-1Sm-0.5Zr alloys. (**a**,**d**) *x* = 4; (**b**,**e**) *x* = 7; (**c**,**f**) *x* = 10.

Figure 2 shows the EBSD maps of solution-treated Mg-xGd-4Y-1Sm-0.5Zr alloys. The solution-treated alloys exhibit equiaxed grains with straight boundaries. When increasing Gd content, the grain size gradually decreases, and the average grain sizes are 95.6 μm, 77.3 μm, and 55.2 μm, respectively. Figure 2d–f present the {0001}, {$10\overset{-}{1}$0}, and $\{11\overset{-}{2}0$} pole figures (PFs) of the solution-treated alloys, indicating that the grains exhibit a random distribution without obvious preferred orientation.

### 3.2. The Microstructure of Hot-Extruded Alloys

**Figure 3:**
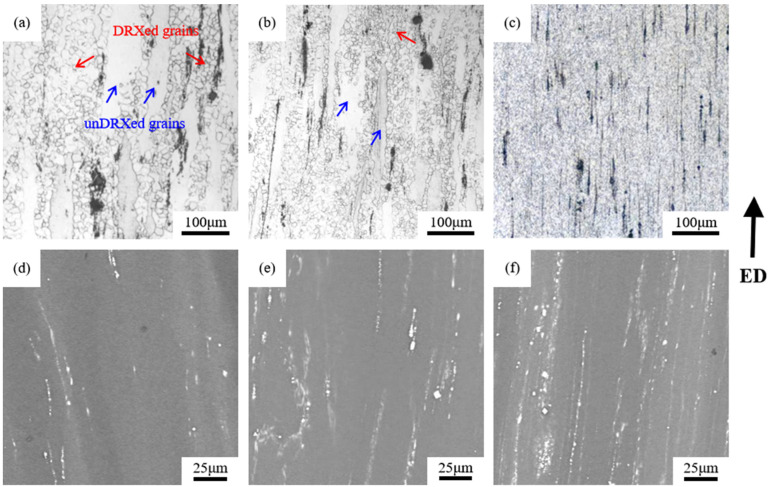
OM images (**a**–**c**) and SEM maps (**d**–**f**) of Mg-*x*Gd-4Y-1Sm-0.5Zr alloys. (**a**,**d**) *x* = 4; (**b**,**e**) *x* = 7; (**c**,**f**) *x* = 10.

Figure 3a–c present the optical micrographs of extruded Mg-*x*Gd-4Y-1Sm-0.5Zr alloys along the extrusion direction (ED). It is shown that the grains of the three alloys have undergone significant refinement, revealing the occurrence of dynamic recrystallization (DRX) during the hot extrusion process. This is attributed to the fact that the hexagonal close-packed (HCP) structure of magnesium alloys results in low stacking fault energy and weak dislocation extension ability, making it difficult to eliminate dislocations through cross-slip or climb. Dislocations generated during deformation rapidly accumulate to form high dislocation density regions, which provide the driving force for DRX, thereby facilitating the occurrence of dynamic recrystallization behavior [21,22,23].

Interestingly, the microstructures of the extruded GWS441 and GWS741 alloys exhibit a bimodal structure, consisting of fine dynamically recrystallized grains and original deformed grains that did not undergo dynamic recrystallization. The deformed grains, as indicated by the arrows in the figures, are elongated along the extrusion direction. Such a bimodal grain structure has been observed in many studies. Tang et al. [24] investigated the effect of initial grain size on the evolution of bimodal grain structure in hot-extruded alloys and proposed that the fraction of un-DRXed grains in the bimodal microstructure is influenced by the initial grain size and increases with the increase in initial grain size.

In addition, black strip-like structures were observed in the hot-extruded alloys, distributed in a streamline pattern along the hot extrusion direction, as indicated by the black arrows in Figure 3a–c. This is because during the hot extrusion process, the undissolved eutectic phases were crushed into numerous intermetallic particles, which aligned along the extrusion direction. These features are more clearly visible in the SEM micrographs of Figure 3d–f.

**Figure 4:**
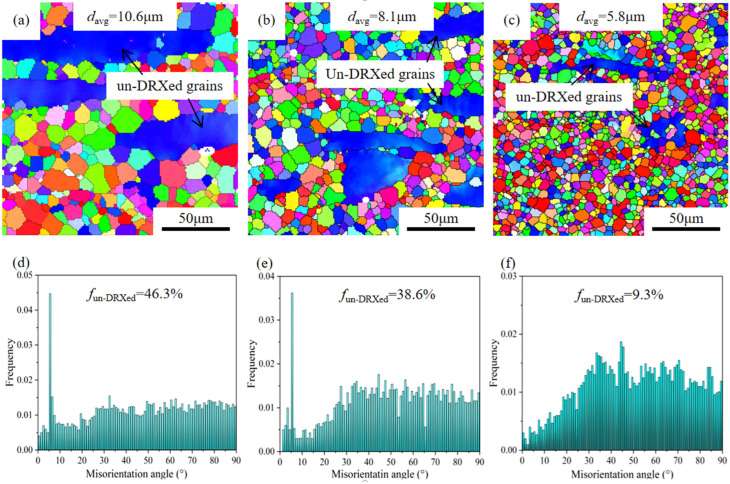
IPF maps (**a**–**c**) and misorientation images (**d**–**f**) of hot extrusion Mg-*x*Gd-4Y-1Sm-0.5Zr alloys. (**a**,**d**) *x* = 4; (**b**,**e**) *x* = 7; (**c**,**f**) *x* = 10.

Figure 4a–c show the inverse pole figure (IPF) maps of the hot-extruded alloys along the extrusion direction (ED). It is evident from the figures that the degrees of DRX vary among the three hot-extruded alloys. The GWS441 alloy exhibits the lowest DRX degree, with the fractions of un-DRXed regions measured to be approximately 46.3%. With the increase in Gd content, the proportion of low-angle grain boundary (LAGB) decreases, the residual dislocation decreases, and the degree of DRX increases gradually. The hot extruded GWS1041 alloy undergoes almost complete DRX, with the proportion of un-DRXed grains decreasing to 9.3% (Figure 4d–f). Obviously, the proportion of un-DRXed regions decreases with the increase in Gd content. In other words, the higher content of the Gd element is beneficial for promoting the DRX of the Mg-*x*Gd-4Y-1Sm-0.5Zr alloy during the hot extrusion process. The average grain sizes of hot-extruded GWS441, GWS741, and GWS1041 alloys are 10.6 μm, 8.1 μm, and 5.8 μm, respectively.

The research shows that Zener–Hollomon (*Z*) is a widely used parameter for analyzing the grain size of hot-deformed alloys, which affects the DRX grain size [25]. Guan et al. [25] set up the Formula (1):

(1) $$Zd_{DRX}^{p}\;=\;\mathrm{constant}$$

where *Z* is the Zener–Hollomon parameter, *d_DRX_* is the average size of DRXed grains, and *p* is the relevant exponent, respectively.

Xu et al. [26] propose a computing Formula (2) for the *Z* parameter:

(2) $$Z\;=\;\overset{•}{\varepsilon }\exp\left(\frac{Q}{RT}\right)$$

where *Q* represents the activation energy for lattice diffusion, $\dot{\varepsilon }$ is the strain rate, *T* is the processing temperature, and *R* is the gas constant.

The research shows that the *Q* value of pure magnesium is 135 KJ/mol [26], and rare earth elements can significantly improve the *Q* value of Mg alloys. Xia et al. [27] calculated the *Q* value of Mg-8.1Gd-4.5Y-0.3Zr alloy as 192 KJ/mol using a hot compression simulation experiment. Wu et al. [28] found that increasing the Gd content can significantly improve the *Q* value of Mg-*x*Gd-0.5Zr alloys. Obviously, according to Formula (2), the increase in Q value can increase the *Z* value, and according to Formula (1), the DRXed grain size decreases with the increase in Z parameter. The results of this experiment also show that the DRXed grain size decreases with the increase in Gd content of the hot-extruded Mg-*x*Gd-4Y-1Sm-0.5Zr alloys.

On the other side, the increase in Z value also increases the dislocation density in the alloy and forms a large number of substructures, thus improving the nucleation rate of DRX [29]. This also explains that DRXed grains increase with Gd content increasing, and the proportion of the DRXed region almost reaches 95% of the hot extrusion GWS1041 alloy.

### 3.3. Age-Hardening and Microstructure Analysis of Peak-Aged Mg-xGd-4Y-1Sm-0.5Zr Alloys

#### 3.3.1. Age-Hardening Analysis

**Figure 5:**
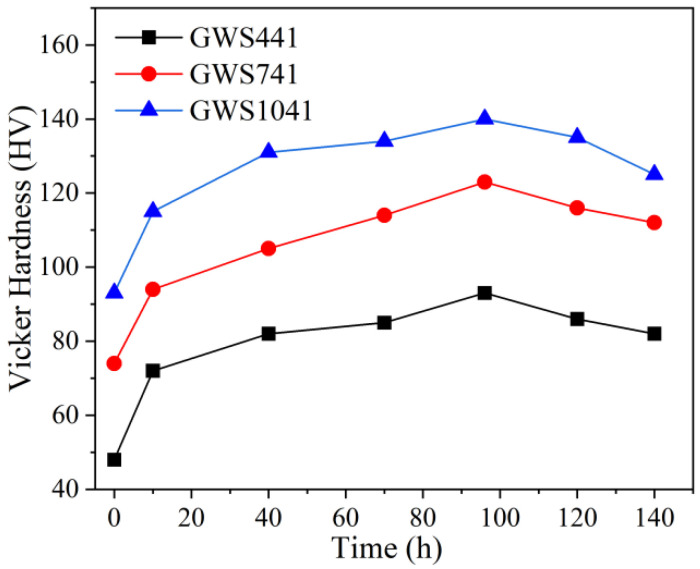
Age hardening curves of hot extrusion Mg-*x*Gd-4Y-1Sm-0.5Zr (*x* = 4, 7, 10) alloys at 200 °C.

For Mg-Gd-Y-Zr series alloys, the aging temperature is typically in the range of 175–250 °C [30]. Previous studies by our research group have revealed that the age-hardening effect of hot-extruded Mg-*x*Gd-4Y-1Sm-0.5Zr alloys is more significant at 200 °C. The age-hardening curves of the three hot-extruded alloys are shown in Figure 5. The hardness of all three alloys initially increases and then gradually declines as the aging time extends. It is evident that after 96 h of aging, all three alloys reach their hardness peaks, and as Gd content rises, the peak hardness also progressively increases. The microstructural characteristics of the three peak-aged alloys are selected for HADDF-STEM analysis in Section 3.3.2.

#### 3.3.2. The Microstructure Analysis of Peak-Aged Mg-*x*Gd-4Y-1Sm-0.5Zr Alloys

**Figure 6:**
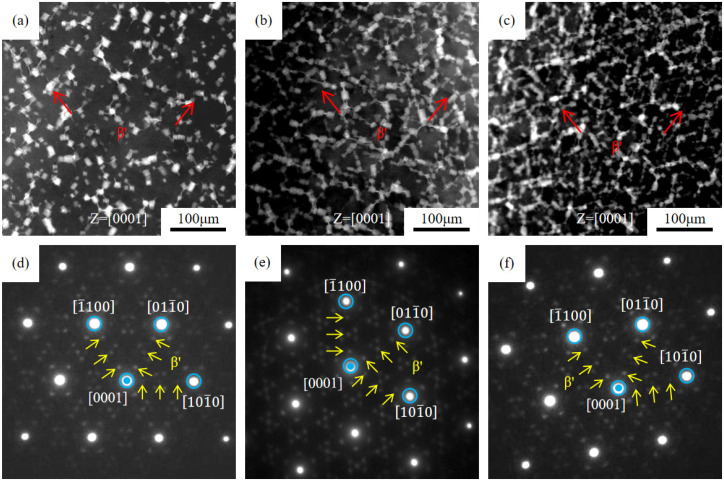
HADDF-TEM (**a**–**c**) and SAED spectrum (**d**–**f**) of hot-extrusion aged Mg-*x*Gd-4Y-1Sm-0.5Zr alloys. (**a**,**d**) *x* = 4; (**b**,**e**) *x* = 7; (**c**,**f**) *x* = 10.

Figure 6 shows the HADDF images and the corresponding selected area electron diffraction (SAED) spectrum of the hot extrusion peak-aged alloys. A large number of white precipitates are distributed in the matrix and are distributed in an approximate network. The corresponding SAED spectrum is shown in Figure 6d–f, and the diffraction axis is parallel to the [0001]α crystal direction. The brighter spots are from the α-Mg matrix, and the weaker diffraction spots are distributed at 1/4$\{10\overset{-}{1}0\}\alpha$, 2/4$\{10\overset{-}{1}0\}\alpha$, and 3/4$\{10\overset{-}{1}0\}\alpha$ positions in these images. Through simulation, the weaker spots are from the β′ phase, with $\left(110\right)\beta^{\prime }//\left(0001\right)\alpha$, $(1\overset{-}{1}1)\beta^{\prime }//(11\overset{-}{2}0)\alpha$ [31,32,33]. The SAED spectrum also shows that the precipitate is mainly β′ phase. Figure 6a–c also illustrate that the amount of β′ increases significantly because of Gd content.

**Figure 7:**
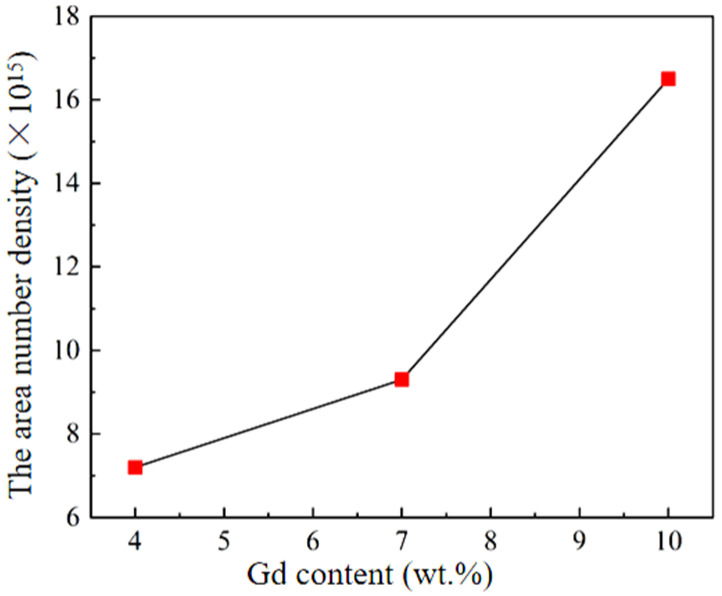
The area number density statistical chart of β′ precipitate in the peak-aged alloys.

Figure 7 shows the area number density of β′ precipitates of the peak-aged alloys. The area number density of β′ phase in GWS441 alloy is the lowest, with 7.1 × 10^15^/m^2^, while the area number density of GWS1041 alloy is the largest, 16.5 × 10^15^/m^2^. Although the time and temperature of aging treatment are different from those reported by Wang [34], all the area number densities of β′ are similar, indicating that the three alloys have reached the peak aging state.

It is obvious that the area number density of the β′ phase increases with increasing Gd. It can be inferred that the β′ phase is composed of Gd-rich components in the specimen, which resulted in higher precipitate density with the increase in Gd content. Wang et al. [35] studied the binding value of stable phases formed between Mg-Mg, Mg-Y, Mg-Gd, Gd-Y, and Gd-Gd atoms in the Mg-Gd-Y alloy. The results showed that the Gd-Y clusters are preferentially formed at the beginning of aging treatment, and these clusters would be transformed into the β′ phase. Because of increasing Gd content, the quantity of Gd-Y clusters increases, and the area number density of β′ precipitates also increases. This result is similar to that of Rong et al. [36], with Gd content increasing, the area number density of β′ gradually increases.

**Figure 8:**
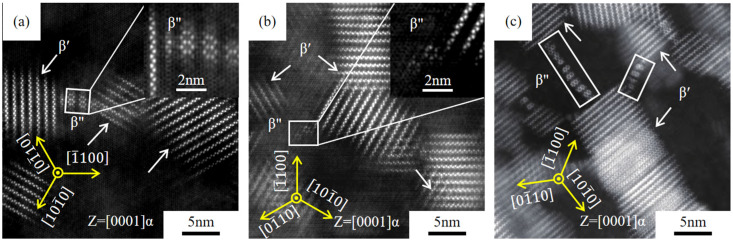
HADDF-STEM images at the $\left[0001\right]\alpha$ axis of hot-extrusion-aged Mg-*x*Gd-4Y-1Sm-0.5Zr alloys. (**a**) *x* = 4; (**b**) *x* = 7; (**c**) *x* = 10.

In addition to the β′ phase, there are some hexagonal precipitates in the three peak-aged alloys, which are distributed between β′ precipitates in the white rectangle of Figure 8a–c. Zhou et al. [37] found that this “tentacle” precipitate is the β″ phase, which precipitates along the $\langle 10\overset{-}{1}0\rangle \alpha$ direction, which is conducive to the precipitation of Gd atoms. Matsuoka et al. [38] believed that the crystal orientation of the β″ phase is related to the orientation of the β′ phase. Xie et al. [39] analyzed the evolution of aging precipitation of Mg-Gd-Y alloy, found that the β″ phase would change into the β′ phase during isothermal aging, and established a transformation model, pointing out that the β′ phase is to reduce elastic strain and achieve faster growth through the β″ phase with a lower nucleation barrier. It can also be found that the β′ phase is formed along the $\langle 10\overset{-}{1}0\rangle \alpha$ crystal direction, in the rectangular region of Figure 8b, which also shows that the β′ phase is transformed from these “tentacle” structures. The research shows that both β″ and β′ phases belong to mainly strengthening phases in Mg-Gd-Y series alloys [40,41,42].

**Figure 9:**
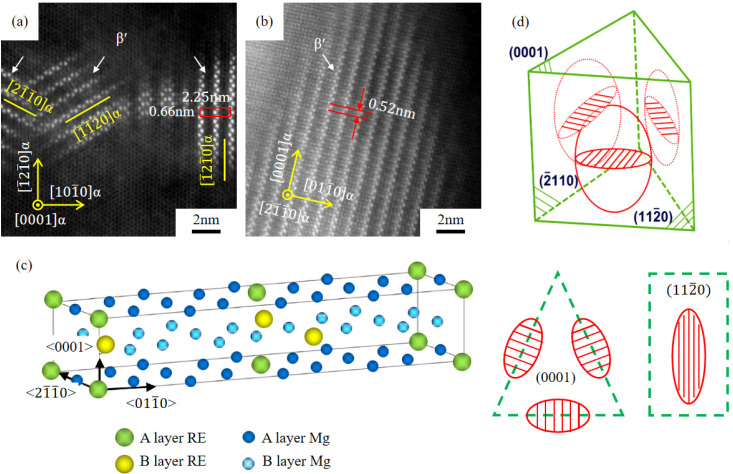
HADDF-STEM of β′ at the [0001]α axis and $[11\overset{-}{2}0]\alpha$ axis (**a**,**b**), schematic diagram of β′ cell (**c**), and schematic diagrams showing the β′ phase in $\left\{0001\right\}\alpha$ and $\{2\overset{-}{1}\overset{-}{1}0\}\alpha$ planes (**d**) of peak-aged GWS441 alloy.

It is obvious that the area number density of the β′ phase is the largest of the three alloys, which is much more than that of the β″ phase (Figure 8a–c), and the β′ phase is the main precipitate in this paper. Figure 9a,b show the HAADF-STEM images of GWS441 alloy at the peak-aged state in both $\langle 0001\rangle \alpha$ and $\langle 2\overset{-}{1}\overset{-}{1}0\rangle \alpha$ orientations. The lattice size of β′ is a ≈ 0.66 nm = 2aMg, b ≈ 2.25 nm = 8d{10-10}Mg, and c ≈ 0.52 nm = cMg, respectively. The three-dimensional atomic distribution model of the β′ phase is shown in Figure 9c.

Figure 9a,b also shows that the β′ phase precipitates in the $(11\overset{-}{2}0)\alpha$, $(\overset{-}{1}2\overset{-}{1}0)\alpha$ and $(2\overset{-}{1}\overset{-}{1}0)\alpha$ crystal planes. The HAADF-STEM image at the $[2\overset{-}{1}\overset{-}{1}0]\alpha$ axis shows that the β′ phase is in a long strip shape, and the long axis is parallel to 〈0001〉α. Figure 9d shows the distribution diagram of the β′ phase in these crystal planes, and it is evident that the β′ phase is distributed in a disk shape. Research believes that the disk-shaped phase precipitated in cylindrical grain planes of the Mg alloys has the greatest blocking on $\left\{0001\right\}\langle 11\overset{-}{2}0\rangle \alpha$ slip, which could significantly improve the precipitation strengthening [43].

#### 3.3.3. Analysis of Mechanical Properties

**Figure 10:**
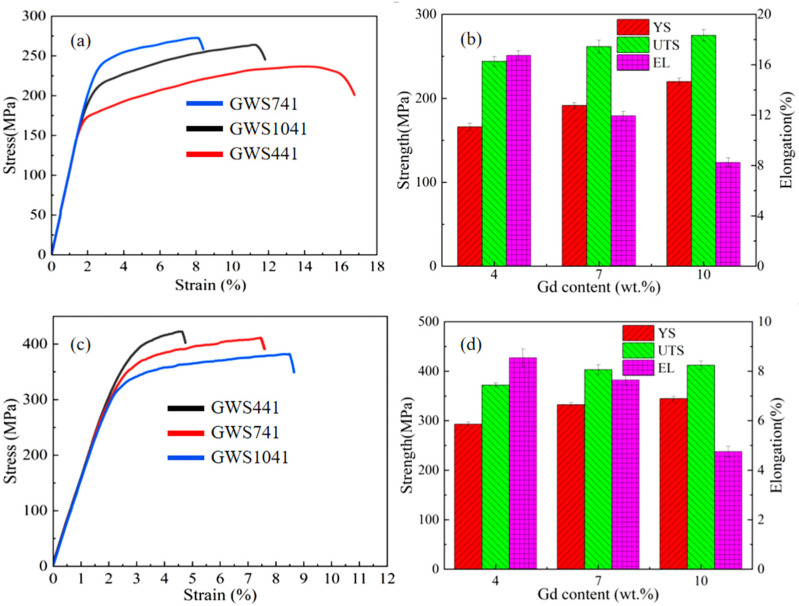
Tensile stress–strain curves and corresponding mechanical properties of hot extruded state (**a**,**b**) and peak aged state (**c**,**d**) Mg-*x*Gd-4Y-1Sm-0.5Zr alloys.

Figure 10 is tensile stress–strain curves and corresponding specific property parameters of Mg-*x*Gd-4Y-1Sm-0.5Zr alloys at the hot extruded state and hot-extruded peak-aged state. It is not difficult to find that the hot extruded GWS441 alloy has the lowest yield strength (YS) and ultimate tensile strength (UTS), which are 166 MPa and 245 MPa, respectively, but shows good elongation, up to 16.8% (Figure 10a). With the increase in Gd content, the YS value (from 166 MPa to 220 MPa) and UTS value (from 245 MPa to 275 MPa) of hot extruded alloy increase, while the elongation (EL) decreases gradually (Figure 10b). Compared with the hot extruded alloy, the YS and UTS of the peak-aged alloy are significantly improved. With Gd content increasing, YS increases from 287 MPa to 345 MPa, UTS increases from 365 MPa to 418 MPa, and EL decreases from 8.5% to 4.2% (Figure 10c,d). It is obvious that the peak-aged GWS741 alloy has the best comprehensive mechanical properties, with the YS, UTS, and EL being 332 MPa, 409 MPa, and 7.8%.

The strengthening mechanisms mainly include the solid solution strengthening (Δ*σ_SS_*), grain boundary strengthening (Δ*σ_GB_*), dislocation strengthening (Δ*σ_DIS_*), and precipitation strengthening (Δ*σ_P_*) [44,45]. The remarkable microstructure characteristic is grain refinement for the hot-extruded alloys, compared with the solid solution alloys. Therefore, with Gd content increasing, the average grain size gradually decreases, and the YS of hot extruded alloy increases gradually. After hot extrusion, the number of grain boundaries increases, resulting in grain boundary strengthening (Δ*σ_GB_*). The Hall–Petch formula [46] describes the relationship between yield strength and grain size, as shown in Formula (3)

(3) $$∆\sigma_{\mathrm{GB}}\;=\;Kd^{-1/2}$$

where *K* is the yield constant and *d* is the average grain diameter. Formula (3) indicates that Δ*σ_GB_* increases with *d* decreasing, which is directly proportional to *d*^−1/2^. Therefore, the method of grain refinement can effectively improve the yield strength of Mg alloys.

The YS of the hot extruded peak-aged alloy is further raised than that of the hot-extruded alloys. It is mainly due to precipitation strengthening (Δ*σ_P_*) from the strengthening precipitates of the peak-aged alloys. Δ*σ_P_* can be calculated by Formula (4) [47]:

(4) $${∆\sigma }_{p=}\frac{Gb}{2\pi \sqrt{1-v}(0.825\sqrt{\frac{d_{t}t_{t}}{f}}\;-0.393d_{t}-0.886t_{t})}ln\frac{0.886\sqrt{d_{t}t_{t}}}{b}$$

where *G* is the shear modulus of magnesium, *f* is the volume fraction of the precipitate, *t*_t_ is the thickness of the disk-shaped phase, *b* is the basal dislocation Burgers vector, *d*_t_ is the average diameter of the disk-shaped phase, and ν is Poisson’s ratio.

Formula (4) shows that Δ*σ_P_* is related to the plane diameter and volume fraction of precipitates, and the volume fraction is proportional to the area number density. Generally, the smaller the grain size, the greater the area number density of the strengthening precipitate, and the higher the Δ*σ_P_*. In the hot extrusion-aged alloys, the size of the β′ phase changes little, but the area number density increases gradually with increasing Gd. It also explains that the reason for the increase in the Δ*σ_P_* value is due to the increase in the area number density of the β′ phase with increasing Gd content.

However, it is puzzling that the UTS of peak-aged GWS1041 alloy is only 9 MPa larger than that of the peak-aged GWS741 alloy, but the EL decreases significantly, only 4.2%. It is evident that there are many streamlined broken second phases in hot extrusion GWS1041 in Figure 3f, which will form a source of stress concentration after aggregation. The broken second phases will easily become the crack initiation point under the action of external load, reducing the mechanical properties of the alloy.

#### 3.3.4. Tensile Fracture Morphology Analysis

**Figure 11:**
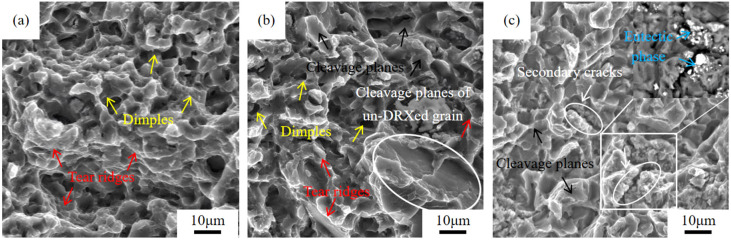
SEM fracture morphology of hot extrusion peak-aged Mg-*x*Gd-4Y-1Sm-0.5Zr alloys. (**a**) *x* = 4; (**b**) *x* = 7; (**c**) *x* = 10.

Figure 11 reveals the SEM morphology of room temperature tensile fracture with peak-aged Mg-*x*Gd-4Y-1Sm-0.5Zr alloys. The fracture morphology with different Gd content shows different fracture characteristics, but the main fracture mechanism is quasi-cleavage fracture. The tensile fracture of GWS441 alloy is mainly composed of dimples, tearing ridges, and cleavage facets, and the dimples are deep (Figure 11a). The main difference between the fracture morphology of GWS741 alloy and GWS441 alloy is that the number of cleavage facets increases significantly, the dimples are fewer, and the fracture of un-DRXed grain with larger size can also be observed, as shown by the white circle in Figure 11b. The fracture morphology of GWS1041 alloy is mainly composed of many cleavage facets (Figure 11c). In addition, secondary cracks are found, which mainly originate from the enrichment of the eutectic phase, as shown by blue arrows in Figure 11c. These secondary cracks, as the source of stress concentration, will accelerate the main crack propagation and significantly reduce the strength. This also proved that the elongation of peak-aged GWS1041 alloy decreased significantly.

### 3.4. Deformation Behavior of Hot Extrusion Peak Aged GWS741 Alloy

**Figure 12:**
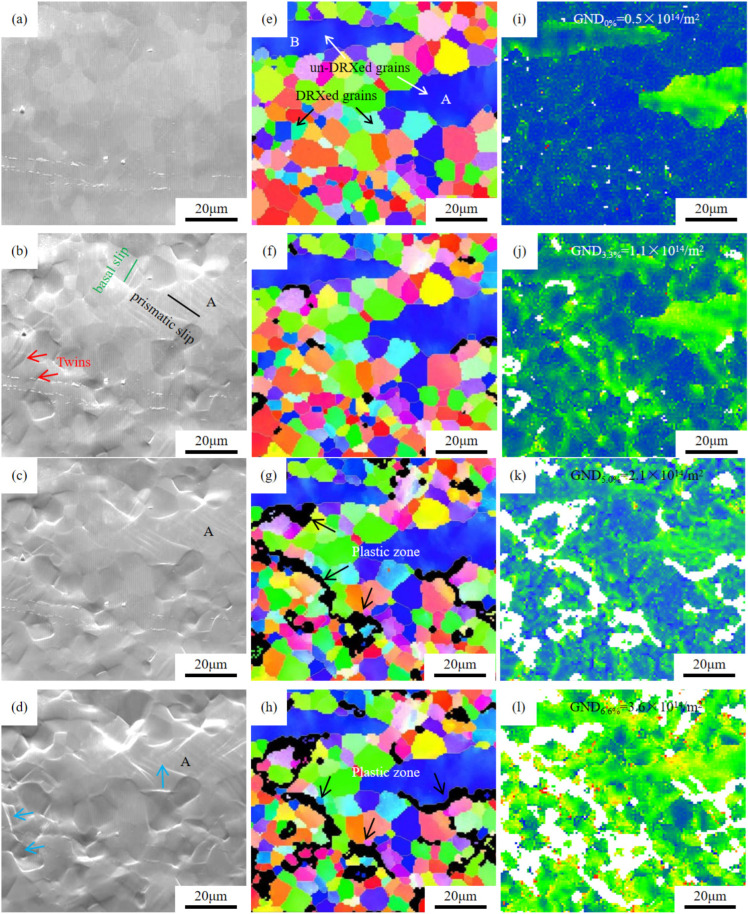
SEM images (**a**–**d**), IPF maps (**e**–**h**), and GND figures (**i**–**l**) of peak aged GWS741 alloy during the in situ tension process. (**a**,**e**,**i**) 0% strain; (**b**,**f**,**j**) 3.3% strain; (**c**,**g**,**k**) 5.0% strain; (**d**,**h**,**l**) 6.6% strain.

Figure 12 shows the deformation behavior of hot-extrusion peak-aged GWS741 alloy during in situ tensile deformation. Figure 12a,e show the SEM images and IPF maps before in situ tension, and the alloy structure consists of un-DRXed deformed grains A, B, and DRXed grains.

When the strain is 3.3%, the obvious slip band is formed in the larger deformed grain A, and the prismatic slip ($\{10\overset{-}{1}0\}\langle 11\overset{-}{2}0\rangle$) occurs. Some twins and basal slip $\left(\{10\overset{-}{1}0\}\langle 11\overset{-}{2}0\rangle \right)$ occurs in a small amount of DRXed grains (Figure 12b). When the strain increases to 5.0%, there is still no deformation in the smaller un-DRXed grain B, but the plastic deformation in grain A continues to accelerate (Figure 12c). And the internal dislocations will gradually accumulate at the grain boundary, form a dislocation pile-up, and lead to the concentration of stress. These accumulated stresses and dislocation pile-up will be further transferred to the interface area between coarse and fine grains, by the black arrow of Figure 12g,h. As the strain is 6.6%, DRXed grain A and twins are deformed greatly, as shown by the blue arrows in Figure 12d. However, there are still no micro-cracks in the observation area, showing better plastic. This is because the large-sized coarse grains can accommodate more plastic deformation and are not easy to crack, which is useful to improve the global plasticity. Zhang et al. [48] investigated the tensile deformation process of ZK60 alloy featuring a bimodal microstructure and discovered that the coarse deformed grains predominantly accounted for the plastic deformation.

In addition, the dislocation movement in the coarse grain is relatively easy, and it is not easy to form a strong dislocation pile-up and back stress hardening, which makes the coarse grain more prone to plastic flow during deformation. Figure 12i–l show the distribution of geometrically necessary dislocation (GND) density under different strains, indicating that the GND density gradually increases as strain increases. When the strain is 6.6%, GND density is 3.6 × 10^14^/m^2^. Ji et al. [49] believe that in a bimodal microstructure, due to the difference in deformation behavior between coarse and fine grains, further accumulation of GND and dislocations will be triggered at the interface, thus forming a back stress hardening effect, which can enhance the integral strength and plasticity of the material.

**Figure 13:**
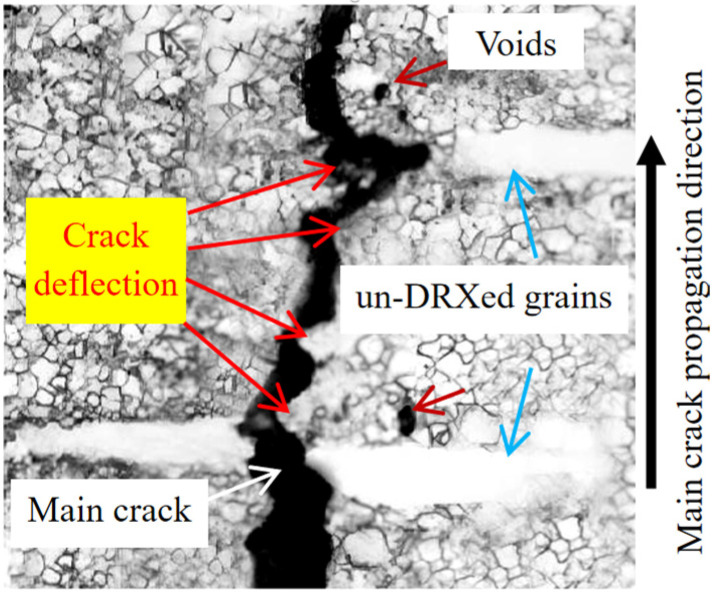
OM figure of peak-aged GWS741 sample along the main crack during tensile failure progress.

Figure 13 illustrates the main crack propagation of peak-aged GWS741 alloy. It can be seen that the main crack propagates through un-DRXed grains, indicating that the propagation of the crack is hindered by the coarse un-DRXed grains, as shown in Figure 13. Many crack deflections can be observed, as shown by the red arrow. It shows a tortuous propagation path of the main crack, and the crack propagation resistance is large, resulting in a large plastic energy consumption, thus improving the fracture toughness during the whole process. Research shows that crack deflection and crack bridging usually reduce the driving force of crack propagation [50,51]. In addition, the tip of the main crack is around the un-DRXed grain, indicating that the crack propagation may be hindered by the un-DRXed grain, which improves fracture resistance.

## 4. Conclusions

1. The grain of hot extruded Mg-*x*Gd-4Y-1Sm-0.5Zr (*x* = 4, 7, 10; wt.%) alloys is refined strongly and forms a bimodal structure. With Gd content increasing, the degree of DRXed increases and average grain size gradually decreases, which are 10.6 μm, 8.1 μm, and 5.8 μm, respectively. Both the fraction of non-dynamic recrystallized grains gradually decreases, with 46.3%, 38.6%, and 9.3%.
2. After aging treatment (200 °C × 96 h), the hot extruded Mg-*x*Gd-4Y-1Sm-0.5Zr alloys reach the peak hardness. As Gd content increases, the area number density of the β′ phase increases with Gd, being 7.1 × 10^15^/m^2^, 9.9 × 10^15^/m^2^, and 16.5 × 10^15^/m^2^, respectively. And the yield strength (YS) increases from 287 MPa to 345 MPa, the ultimate tensile strength (UTS) increases from 365 MPa to 418 MPa, and elongation (EL) decreases from 8.5% to 4.2%. The fracture mechanism is a quasi-cleavage fracture.
3. The hot extrusion peak-aged GWS741 has the optimal performance, and the YS, UTS, and EL are 332 MPa, 409 MPa, and 7.8%, respectively. From the in situ experiment, the coarse un-DRXed grain occurs prismatic ($\{10\overset{-}{1}0\}\langle 11\overset{-}{2}0\rangle$) slip, and the DRXed grains occur basal ($\left\{0001\right\}\langle 11\overset{-}{2}0\rangle$) slip and twin deformation. When the strain is 6.6%, there is still no micro-crack in the bimodal structure region, showing high plasticity. During the tensile failure process, the main crack propagates along a zigzag path, forming the crack deflection, and the main crack passes through the long un-DRXed grain and bypasses the un-DRXed grain.

## Figures and Tables

**Table 1:** The composition of as-cast Mg-*x*Gd-4Y-1Sm-0.5Zr (*x* = 4, 7, 10) (%).

	Gd	Y	Sm	Zr
Mg-4Gd-4Y-1Sm-0.5Zr (GWS441)	3.95	3.88	0.91	0.51
Mg-7Gd-4Y-1Sm-0.5Zr (GWS741)	6.91	3.75	0.87	0.48
Mg-10Gd-4Y-1Sm-0.5Zr (GWS1041)	9.87	3.92	0.94	0.46

## Data Availability

The original contributions presented in this study are included in the article. Further inquiries can be directed to the corresponding author.
